# Association between decreased thyroid stimulating hormone and hyperuricemia in type 2 diabetic patients with early-stage diabetic kidney disease

**DOI:** 10.1186/s12902-020-00672-8

**Published:** 2021-01-06

**Authors:** Xiaomeng Feng, Jing Huang, Yan Peng, Yuan Xu

**Affiliations:** 1 Department of Endocrinology, Beijing Chao-Yang Hospital, Capital Medical University, Beijing, 100020 China; 2 Department of Nephrology, Beijing Chao-Yang Hospital, Capital Medical University, Beijing, 100020 China; 3 Department of Nutrition, Liangxiang Hospital of Beijing, Fangshan District, Beijing, 102400 China

**Keywords:** Type 2 diabetes mellitus, Diabetic kidney disease, Uric acid, Thyroid hormones

## Abstract

**Background:**

Serum uric acid (SUA) is associated with the development of diabetic kidney disease (DKD). Thyroid hormones can regulate metabolism and insulin resistance. The relationship between SUA and thyroid function in patients with DKD is still uncertain. In current study, we aimed to investigate the association between thyroid stimulating hormone (TSH) and SUA in type 2 diabetic patients with early-stage DKD.

**Methods:**

Two hundred fifty-four type 2 diabetic patients with early-stage DKD were enrolled in current study and were further classified as high SUA group (SUA level > 420 μmol/L in males or > 360 μmol/L in females, *n* = 101) and normal SUA group (SUA level ≤ 420 μmol/L in males or ≤ 360 μmol/L in females, *n* = 153). Eighty-five control subjects were recruited as control group. The clinical characteristics were obtained via face-to-face surveys and medical records.

**Results:**

Compared with normal SUA group and control group, high SUA group exhibited the increased SUA level, and the decreased TSH level (*P* < 0.017 for all), and no significant difference was detected in SUA and TSH between normal SUA group and control group. TSH was negatively associated with SUA (*r* = − 0.35, *P* < 0.001) in type 2 diabetic participants with early-stage DKD. Furthermore, the decreased TSH level was independently correlated with higher SUA level (*β* = − 25.69, *P* < 0.001), and retained a significant association with hyperuricemia (odds ratio = 1.73, *P* = 0.002) after adjusting for confounding factors in type 2 diabetic patients with early-stage DKD.

**Conclusions:**

TSH is negatively correlated with SUA, and decreased TSH is an independent risk factor for hyperuricemia in type 2 diabetic patients with early-stage DKD. These results indicate that thyroid hormones, TSH in particular, might participate in regulating uric acid metabolism in patients with early-stage DKD.

## Background

The prevalence of type 2 diabetes mellitus (T2DM) is greatly increasing, and many patients suffer from diabetes-related complications. Diabetic kidney disease (DKD), one of the major microvascular complications of diabetes, is the main cause of end-stage renal disease and is associated with high morbidity and mortality.

In human beings, uric acid (UA) is the end product in purine metabolism, and approximately 70% of UA is eliminated through the kidney [1]. The increased serum uric acid (SUA) has been reported to be correlated with the progression of insulin resistance [2], metabolic syndrome [3], and T2DM [4]. Recently, SUA has been documented to cause the development of microvascular diseases and thereby renal injury in DKD by several reported effects, including inducing the endothelial dysfunction [5], causing the inflammatory cascades [6], activating the profibrotic cytokine [7], and increasing the activity of the renin-angiotensin aldosterone system [6]. Clinical research has found that SUA was significantly associated with albuminuria in patients with T2DM [8]. Therefore, the elevated SUA has been regarded as one of the major predictors of DKD [9].

Accumulating evidence has indicated that thyroid hormones can regulate metabolism and insulin resistance [10]. Thyroid dysfunction can worsen glucose metabolism and induce hyperglycemia in patients with T2DM, promoting the risk of diabetic complications. Hyperglycemia decreases the level of thyroid stimulating hormone (TSH) and reduces the conversion of thyroxine to triiodothyronine in the peripheral tissues [11]. There have been several studies about the relationship between SUA and thyroid function in different populations. However, the results were conflicting. Some studies supported that there was a higher risk of hyperuricemia in patients with hypothyroidism [12] or with subclinical hypothyroidism [13] relative in comparison to the general subjects, and there was a negative correlation between SUA and free triiodothyronine (FT3) in participants with low UA contents [14]. On the other hand, a linear relationship between free thyroxine (FT4) and SUA was observed in individuals without overt thyroid dysfunction [15]. Moreover, a recent study has also demonstrated that the decrease of SUA by the treatment of febuxostat or allopurinol led to the increase of TSH in patients with gout [16].

To the best of our knowledge, studies of the relationship between SUA and thyroid hormones in patients with DKD have been few in number, with incomplete information regarding the underlying interaction, although there have been some researches with controversial results to explore this association in different populations. Additionally, there is collinearity among thyroxine, triiodothyronine and TSH, and TSH is the most sensitive and the earliest changing index in thyroid hormones. Therefore, in present study, we aimed to investigate the possible association between TSH and SUA in type 2 diabetic patients with early-stage DKD.

## Methods

### Study population

The study protocol was approved by the Medicine and Pharmacy Ethics Committee of Beijing Chao-Yang Hospital, Capital Medical University. Written informed consent was collected from each subject.

The participants aged from 18 to 80 years were recruited consecutively from May 2017 to March 2019 in a group of outpatients at the Department of Endocrinology, Beijing Chao- Yang Hospital, Capital Medical University, Beijing, China.

Patients were diagnosed with T2DM according to the World Health Organization (WHO) criteria, and were diagnosed with early-stage DKD as defined by the consensus on DKD by Chinese Medical Association as urinary albumin-to-creatinine ratio (UACR) in 30 ~ 300 mg/g [17]. Subjects were selected in line with both results of UACR from two urine samples collected on different days in 1 month were in 30 ~ 300 mg/g. Hyperuricemia was defined as the SUA level > 420 μmol/L in males or > 360 μmol/L in females [18].

People with primary glomerulonephritis or kidney diseases caused by secondary conditions other than diabetes, with gout or medicines that influence UA metabolism (including diuretics, sodium-dependent glucose transporter 2 inhibitors, UA lowering agents, etc.), with history of thyroid diseases or medicines that influence thyroid function, with infection, malignancies, autoimmune disease, hypertension, cardiovascular disease or pregnancy, and drinking alcohol in last 2 weeks were excluded from all groups.

People with type 1 diabetes or other specific types of diabetes as defined by the WHO classification of diabetes mellitus were excluded from the study. Moreover, people with recent acute diabetic complications, including ketoacidosis, hyperosmolar non-ketotic diabetic coma and lactic acidosis, were also excluded from the study. In addition, none of the control participants had a history of glycometabolism abnormality.

Two hundred fifty-four type 2 diabetic patients with early-stage DKD were enrolled for the study, and were further divided into high SUA group (*n* = 101) and normal SUA group (*n* = 153). Eighty-five control subjects were recruited as control group.

### Measurements of clinical parameters

The subject’ health status and medical history were obtained using a standard questionnaire (see Additional file 1) and medical records via the face-to-face surveys. Subjects wore only underwear for weight and height measurements. Body mass index (BMI) was determined using weight (kg) / [height (m)]^2^. Blood pressures were collected from the non-dominant arm after 5 min of subjects sitting using a same calibrated standard mercury sphygmomanometer.

Three tubes of serum [3 ml for each tube, for total cholesterol (TC), high-density lipoprotein cholesterol (HDL-C), low-density lipoprotein cholesterol (LDL-C), triglycerides (TG), fasting blood glucose (FBG), creatinine (CR) and SUA, for fasting insulin (FINS), and for FT3, FT4 and TSH, respectively] and one tube of plasma [2 ml, for glycated hemoglobin (HbA1c)] from each subject were collected in the morning after an 8-h overnight fast. Random spot urine samples were obtained after 24 h without physical exercise. UACR was determined as the mean of measurements of two urine samples obtained on different days in 1 month.

FBG level was determined by the hexokinase method, TG level was determined by the glycerol phosphate oxidase-peroxidase method, HDL-C and LDL-C levels were determined by the direct method, TC and serum CR levels were determined by the enzymatic method, and SUA level was determined by the uricase method, using a Dade-Behring Dimension RXL Autoanalyzer (Dade Behring Diagnostics, Marburg, Germany). FINS was measured by the electrochemiluminescence method using a Beckman Access 2 (Fullerton, CA, USA). HbA1c was measured by high-performance liquid chromatography on a HLC-723G7 analyzer (Tosoh Corporation, Japan). Insulin resistance was calculated with the equation: homeostasis model assessment of insulin resistance (HOMA-IR) = FINS (mIU/L) × FBG (mmol/L) / 22.5. The estimated glomerular filtration rate (eGFR) was calculated using the Chinese modification of diet in renal disease (MDRD) equation: eGFR (ml/min/1.73 m^2^) = 175 × (serum creatinine) ^-1.234^ × (age)^-0.179^ × (0.79 for female) [14]. Urinary creatinine was detected by enzymatic method (BNProSpec, SIEMENS, Germany); urinary albumin was measured by scattering turbidimetry (Abbot c16000, Abbott Diagnostics, IL, USA). FT3, FT4 and TSH were measured by electrochemiluminescence immunoassay using an Abbott Architect i2000 (Abbott Diagnostics, IL, USA). The reference intervals for FT3, FT4 and TSH were 2.30 ~ 4.20 pg/ml, 0.89 ~ 1.76 ng/dl and 0.55 ~ 4.78 μIU/ml, respectively.

All operations were performed by the same well-trained group, and all obtained data were inspected by the principal investigator. Blood and urine analyses were conducted twice in the Central Laboratory of Beijing Chao-Yang Hospital, Capital Medical University, and finally the average values were taken. The factors that might interfere with the measurement results were governed by the laboratory technologists. The laboratory reagents and instruments were inspected using the quality control samples before the samples of participants were run. Moreover, if the findings were outside the reference intervals, the measurements would be checked and repeated by laboratory technologists.

### Statistical analysis

All data were analyzed using Statistical Package for Social Sciences version 20.0 (SPSS, Inc., Chicago, IL, USA). The normality of the data distribution was checked by the Kolmogorov-Smirnov test. Normally distributed data were given as the means ± standard deviations, and non-normally distributed data were expressed as medians with 25th and 75th percentiles. Comparisons of the clinical parameters in three groups were analyzed by one-way ANOVA (normally distributed data) or Kruskal-Wallis H test (non-normally distributed data). Proportions were analyzed by the chi-squared test. Since TSH was non-normally distributed, the association between TSH and SUA in type 2 diabetic patients with early-stage DKD was examined using Spearman’s correlation coefficient analysis. Hierarchical multivariate linear and logistic regressions were adopted, adjusting for established and potential confounding factors, to explore the relationship between TSH and SUA, and the correlation between TSH and hyperuricemia in type 2 diabetic patients with early-stage DKD. The basic adjusted model adjusted for age and gender, and the fully adjusted model additionally adjusted for BMI, SBP, TG, FBG, FINS, eGFR, and UACR. All statistical tests were two-tailed, and a *P*-value < 0.05 was considered as statistical significance for the findings. However, *P* < 0.017 (0.05 divided by the times of comparison) was used to indicate statistical significance for the multiple comparison.

## Results

### Clinical characteristics of study participants in three groups

**Fig. 1:**
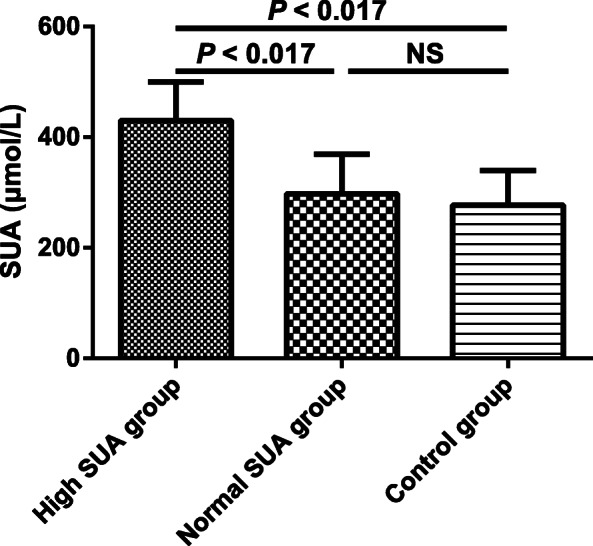
SUA level measured in all study participants. The values are expressed as the means ± standard deviations. High SUA group, subjects with hyperuricemia in type 2 diabetic patients with early-stage diabetic kidney disease (*n* = 101); Normal SUA group, subjects with normal SUA level in type 2 diabetic patients with early-stage diabetic kidney disease (*n* = 153); Control group, control subjects (*n* = 85); SUA, serum uric acid

**Fig. 2:**
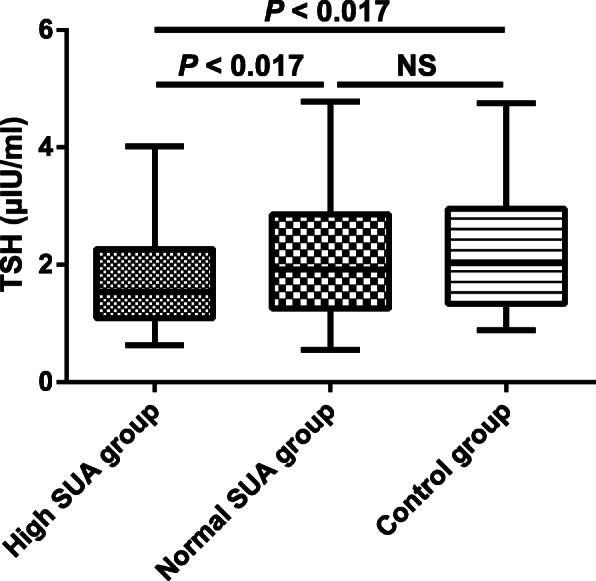
TSH level measured in all study participants. The values are expressed as the medians (25th and 75th percentiles). High SUA group, subjects with hyperuricemia in type 2 diabetic patients with early-stage diabetic kidney disease (*n* = 101); Normal SUA group, subjects with normal SUA level in type 2 diabetic patients with early-stage diabetic kidney disease (*n* = 153); Control group, control subjects (*n* = 85); TSH, thyroid stimulating hormone

The study cohort included 254 type 2 diabetic participants with early-stage DKD consisting of high SUA group and normal SUA group, and 85 control participants as control group. The clinical characteristics of all study participants were listed in Table 1. The participants in three groups were similar in gender, age, systolic blood pressure (SBP), diastolic blood pressure (DBP) and FT3 (*P* > 0.05 for all). A significant trend was observed for BMI, TC, HDL-C, LDL-C, TG, FBG, HbA1c, FINS, HOMA-IR, CR, eGFR, UACR, FT4, TSH and SUA in three groups (*P* < 0.01 for all). High SUA group had the significantly increased levels of BMI, TG, FBG, HOMA-IR, CR and FT4, and the significantly decreased level of eGFR compared with normal SUA group and control group (*P* < 0.017 for all). The levels of BMI, TG, FBG and HOMA-IR were significantly elevated in normal SUA group than those in control group (*P* < 0.017 for all), and no significant difference was detected in CR, eGFR and FT4 between normal SUA group and control group (*P* > 0.017 for all). Both high SUA group and normal SUA group exhibited the significantly higher levels of TC, LDL-C, HbA1c, FINS and UACR, and the significantly lower level of HDL-C than control group (*P* < 0.017 for all), and there was no significant difference in these parameters between high SUA group and normal SUA group (*P* > 0.017 for all). Furthermore, compared with normal SUA group and control group, high SUA group was with a higher SUA level (Fig. 1) and a lower TSH level (Fig. 2) (*P* < 0.017 for both)*.* However, no significant difference was observed in SUA and TSH between normal SUA group and control group (*P* > 0.017 for both). The decreased TSH in high SUA group might indicate the possible association between thyroid function and uric acid metabolism in type 2 diabetic participants with early-stage DKD.

**Table 1:** Clinical characteristics of study participants in three groups

Parameters	High SUA group (*n* = 101)	Normal SUA group (*n* = 153)	Control group (*n* = 85)	*P*
Gender (M/F)	57/44	97/56	52/33	0.537
Age (years)	52.54 ± 11.04	53.10 ± 10.89	51.35 ± 10.77	0.494
BMI (kg/m^2^)	27.47 ± 4.83‡†	25.09 ± 4.42†	23.58 ± 3.05	< 0.001
SBP (mmHg)	117.05 ± 9.32	118.89 ± 8.68	118.28 ± 11.82	0.337
DBP (mmHg)	67.85 ± 8.20	68.66 ± 8.02	68.55 ± 7.84	0.718
TC (mmol/L)	4.83 ± 1.27†	4.79 ± 1.35†	4.33 ± 0.61	0.006
HDL-C (mmol/L)	1.03 ± 0.26†	1.09 ± 0.31†	1.54 ± 0.33	< 0.001
LDL-C (mmol/L)	2.78 ± 0.83†	2.76 ± 1.07†	2.39 ± 0.46	0.007
TG (mmol/L)	2.35 (1.51, 2.98)‡†	1.65 (1.12, 2.46)†	0.78 (0.62,1.12)	< 0.001
FBG (mmol/L)	9.72 ± 2.17‡†	7.87 ± 2.57†	5.02 ± 0.34	< 0.001
HbA1c (%)	8.58 ± 1.93†	8.88 ± 2.05†	5.57 ± 0.39	< 0.001
FINS (mIU/L)	15.30 (10.45,19.65)†	15.10 (9.65,18.65)†	8.70 (5.37,12.40)	< 0.001
HOMA-IR	5.51 (4.75, 7.72)‡†	4.68 (3.33, 6.53) †	1.99 (1.26, 2.76)	< 0.001
CR (μmol/L)	74.26 ± 22.61‡†	61.11 ± 14.83	61.97 ± 11.29	< 0.001
eGFR (mL/min/1.73m^2^)	108.66 ± 37.07‡†	133.27 ± 33.97	128.70 ± 30.71	< 0.001
UACR (mg/g)	38.01 (33.16,94.46)†	37.76 (34.08,55.84)†	3.45 (1.79, 5.29)	< 0.001
FT3 (pg/ml)	3.61 ± 0.52	3.50 ± 0.42	3.47 ± 0.37	0.068
FT4 (ng/dl)	1.37 ± 0.19‡†	1.25 ± 0.17	1.23 ± 0.14	< 0.001
TSH (μIU/ml)	1.54 (1.09, 2.27) ‡†	1.92 (1.25, 2.86)	2.03 (1.33, 2.95)	< 0.001
SUA (μmol/L)	420.33 ± 70.05‡†	297.63 ± 71.79	277.10 ± 62.59	< 0.001

**Abbreviations:** *High SUA group* Subjects with hyperuricemia in type 2 diabetic patients with early-stage diabetic kidney disease, *Normal SUA group* Subjects with normal SUA level in type 2 diabetic patients with early-stage diabetic kidney disease, *Control group* Control subjects, *BMI* Body mass index, *SBP* Systolic blood pressure, *DBP* Diastolic blood pressure, *TC* Total cholesterol, *HDL-C* High-density lipoprotein cholesterol, *LDL-C* Low-density lipoprotein cholesterol, *TG* Triglycerides, *FBG* Fasting blood glucose, *HbA1c* Glycated hemoglobin, *FINS* Fasting insulinm, *HOMA-IR* Homeostasis model assessment for insulin resistance, *CR* Creatinine, *eGFR* Estimated glomerular filtration rate, *UACR* Urinary albumin-to-creatinine ratio, *FT3* Free triiodothyronine, *FT4* Free thyroxine, *TSH* Thyroid stimulating hormone, *SUA* Serum uric acid

‡*P* < 0.017 (0.05 divided by the times of comparison), significantly different compared with normal SUA group; † *P* < 0.017 (0.05 divided by the times of comparison), significantly different compared with control group

### Correlation between TSH and SUA in type 2 diabetic participants with early-stage DKD

**Fig. 3:**
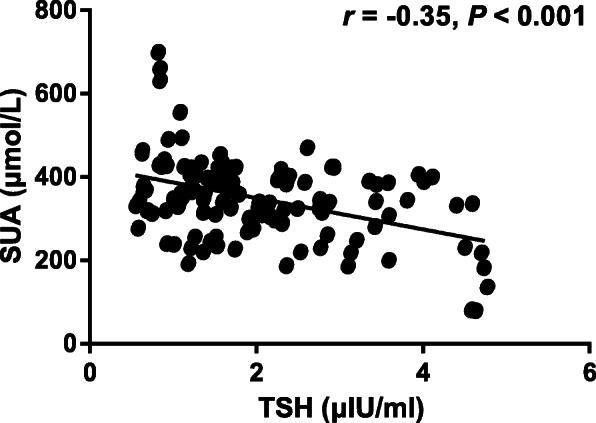
Correlation between TSH and SUA in type 2 diabetic patients with early-stage DKD. TSH, thyroid stimulating hormone; SUA, serum uric acid; DKD, diabetic kidney disease (*n* = 254)

Then, the Spearman’s analysis was performed between SUA and TSH in 254 type 2 diabetic participants with early-stage DKD including individuals in high SUA group and normal SUA group. As exhibited in Fig. 3, SUA was negatively correlated with TSH [*r* = − 0.35, 95% confidence interval (CI): − 0.43 to − 0.21, *P* < 0.001] in type 2 diabetic participants with early-stage DKD, suggesting that decreased TSH was associated with elevated SUA in type 2 diabetic participants with early-stage DKD.

### Multiple regression analysis of TSH correlated with SUA in type 2 diabetic participants with early-stage DKD

Furthermore, to explore the role of TSH in uric acid metabolism, the hierarchical multiple regression analysis was performed to determine whether TSH was independently correlated with SUA in type 2 diabetic participants with early-stage DKD. As presented in Table 2, the results showed that TSH was negatively associated with SUA in unadjusted model (*β* = − 39.52, 95% CI: − 47.35 to − 27.69, *P* < 0.001), and this association remained significant after adjustment for age and gender (*β* = − 34.91, 95% CI: − 44.86 to − 24.95, *P* < 0.001), as well as additionally adjusting for BMI, SBP, TG, FBG, FINS, eGFR, and UACR (*β* = − 25.69, 95% CI: − 33.38 to − 18.00, *P* < 0.001) in type 2 diabetic participants with early-stage DKD.

**Table 2:** Multiple regression of TSH associated with SUA in type 2 diabetic patients with early-stage DKD

Parameters	*β*	SE	95% CI	Standardized *β*	*P*
Model 1^a^	−39.52	4.99	−47.35 ~ − 27.69	−7.52	< 0.001
Model 2^b^	−34.91	5.06	−44.86 ~ − 24.95	−0.40	< 0.001
Model 3^c^	−25.69	3.90	−33.38 ~ − 18.00	−0.29	< 0.001

**Abbreviations:** *SE* Standard error, *CI* Confidence interval, *TSH* Thyroid stimulating hormone, *SUA* Serum uric acid, *DKD* Diabetic kidney disease

The results were based on hierarchical multivariate linear regression taking TSH as a continuous variable in the models (*n* = 254). ^a^Model 1: unadjusted model; ^b^Model 2: basic adjusting model, adjusted for age and gender; ^c^Model 3: fully adjusted model, additionally adjusting for body mass index (BMI), systolic blood pressure (SBP), triglycerides (TG), fasting blood glucose (FBG), fasting insulin (FINS), estimated glomerular filtration rate (eGFR), and urinary albumin-to-creatinine ratio (UACR)

### Logistic regression analysis of TSH associated with hyperuricemia in type 2 diabetic patients with early-stage DKD

Subsequently, to further clarify the role of TSH in uric acid metabolism, the hierarchical logistic regression of the association between TSH and hyperuricemia in type 2 diabetic patients with early-stage DKD was analyzed, and the results were shown in Table 3. The unadjusted odds ratio (OR) for the association between TSH and hyperuricemia was 1.57 (95% CI: 1.21 to 2.04, *P* = 0.001), and the association retained significant after adjustment for age and gender (OR = 1.66, 95% CI: 1.27 to 2.17, *P* < 0.001) in type 2 diabetic patients with early-stage DKD. Notably, decreased TSH with an OR of 1.73 (95% CI: 1.22 to 2.47, *P* = 0.002) was associated with hyperuricemia in type 2 diabetic patients with early-stage DKD after adjustment for age, gender, BMI, SBP, TG, FBG, FINS, eGFR, and UACR.

**Table 3:** Logistic regression of TSH associated with hyperuricemia in type 2 diabetic patients with early-stage DKD

	*β*	SE	OR	95% CI	*P*
Model 1^a^	0.45	0.13	1.57	1.21 ~ 2.04	0.001
Model 2^c^	0.51	0.14	1.66	1.27 ~ 2.17	< 0.001
Model 3^b^	0.55	0.18	1.73	1.22 ~ 2.47	0.002

**Abbreviations:** *SE* Standard error, *OR* Odds ratio, *CI* Confidence interval, *TSH* Thyroid stimulating hormone, *DKD* Diabetic kidney disease

The results were based on hierarchical logistic regression taking TSH as a continuous variable in the models (*n* = 254). ^a^Model 1: unadjusted model; ^c^Model 2: basic adjusted model, adjusting for age and gender; ^b^Model 3: fully adjusted model, additionally adjusting for body mass index (BMI), systolic blood pressure (SBP), triglycerides (TG), fasting blood glucose (FBG), fasting insulin (FINS), estimated glomerular filtration rate (eGFR), and urinary albumin-to-creatinine ratio (UACR)

## Discussion

Hyperuricemia is one of the characteristics of metabolic syndrome which is based on insulin resistance [19]. Metabolic syndrome and insulin resistance might present obesity, hypertriglyceridemia, hyperglycemia, hyperinsulinemia and hyperuricemia [19]. In present study, subjects with hyperuricemia in type 2 diabetic patients with early-stage DKD had increased BMI, TG, FBG, HOMA-IR and SUA values compared to subjects with normal SUA level in type 2 diabetic patients with early-stage DKD and the controls. The levels of BMI, TG, FBG and HOMA-IR were significantly elevated in subjects with normal SUA level in type 2 diabetic patients with early-stage DKD than those in the controls. Type 2 diabetic patients with early-stage DKD in both high SUA group and normal SUA group exhibited the higher levels of TC, LDL-C, HbA1c and FINS, and the lower level of HDL-C than the control subjects. Our findings suggested that type 2 diabetic patients with early-stage DKD were with higher insulin resistance than the controls, and that subjects with hyperuricemia presented further increased insulin resistance compared with subjects with normal SUA level in type 2 diabetic patients with early-stage DKD.

Accumulating evidence has demonstrated that SUA is independently associated with DKD in type 2 diabetic patients [20]. Hyperuricemia plays an important role in the development of DKD, and there is evidence that the reduction in SUA value prevents the progression of DKD [21]**.** In the early state of DKD, eGFR might present a reduction after a momentary elevation, and UACR shows increase; with the development of the renal injury, serum CR will elevate eventually [22]. Our present study indicated that type 2 diabetic patients with early-stage DKD exhibited higher UACR level than control subjects. Participants with hyperuricemia in type 2 diabetic patients with early-stage DKD had the higher CR value, and the lower eGFR value compared with participants without hyperuricemia in type 2 diabetic patients with early-stage DKD and the controls. Our results suggested that patients with hyperuricemia might present more severe renal injury than patients with normal SUA level in type 2 diabetic patients with early-stage DKD, and that hyperuricemia might be associated with DKD.

Importantly, we reported in present study that FT4 was higher, and TSH was lower in patients with hyperuricemia in type 2 diabetic patients with early-stage DKD compared with patients with normal SUA level in type 2 diabetic patients with early-stage DKD and in the controls. TSH was negatively correlated with SUA in type 2 diabetic participants with early-stage DKD. The multiple regression analysis showed that after adjusting for the confounders, the decreased TSH was independently related to the higher SUA in type 2 diabetic patients with early-stage DKD. The logistic regression analysis presented that the reduction of TSH was independently associated with hyperuricemia in type 2 diabetic patients with early-stage DKD. Our findings suggested an association between thyroid function, TSH in particular, and UA metabolism in type 2 diabetic patients with early-stage DKD.

The association between SUA and thyroid function has been assessed in different populations in several previous studies which provided controversial results. An increase in hyperuricemia rates was observed in patients with hypothyroidism [12] and in patients with subclinical hypothyroidism [13] compared to the general individuals, but no relationship between hypothyroidism and hyperuricemia was found in these studies. FT3 was found to be negatively related to SUA in participants with low SUA levels [14]. Moreover, another research reported no relationship between SUA and thyroid hormones in patients with thyroid dysfunction [23]. However, the majority of previous studies are consistent with our findings. A previous study documented that the risk of hyperuricemia was significantly increased in individuals with higher FT4 level [15]. Additionally, a recent study also demonstrated that the decrease of SUA by the treatment of febuxostat or allopurinol exerted effects on the elevation of TSH in patients with gout [16]. These controversial findings might result from the confounding factors of study participants, including race, age, gender, health condition, medical history and so on.

It has been speculated that the negative relationship between SUA and TSH in current research might be due to insulin resistance [24]. Hyperuricemia is one of the manifestations of insulin resistance, and excessive TSH and insufficient thyroxine and triiodothyronine contribute to insulin resistance [25]. There may be a compensatory reduction in TSH (the most sensitive index in thyroid hormones) which leads to the elevation of thyroxine and triiodothyronine, and further contributes to overcome insulin resistance. We hypothesize that the decreased TSH in type 2 diabetic patients with early-stage DKD according to the increase of SUA in our study might have reflected a compensatory result to counterbalance the increased insulin resistance which was indicated by the higher SUA.

There are some limitations in the present study. Firstly, the participants recruited in the current cohort were limited to type 2 diabetic patients with early-stage DKD individuals; hence, our results may not be directly applicable to other subjects. Secondly, the current study was a single center research, and the sample size of the study was relatively small; therefore, the findings of the present research might be influenced by some bias. Thirdly, our study is a cross-sectional study which could not confirm the causal relation but rather suggesting a link between the decrease of TSH and the probability of being hyperuricemia in type 2 diabetic patients with early-stage DKD. Our present research provided the possible hypotheses to be identified and developed by the prospective cohort and mechanistic researches. A follow-up study may be essential to assess whether thyroid function is independently related to hyperuricemia in type 2 diabetic patients with early-stage DKD eventually.

## Conclusions

The current findings suggest that TSH is negatively correlated with SUA, and decreased TSH is an independent risk factor for hyperuricemia in type 2 diabetic patients with early-stage DKD. These results indicate that thyroid hormones, TSH in particular, might participate in regulating uric acid metabolism in patients with early-stage DKD. Furthermore, since hyperuricemia has been considered as a risk factor of DKD, our results suggest that thyroid hormones may also play a key role in the development of DKD, indicating that prevention and treatment of DKD may benefit from the management of thyroid function.

## Supplementary Information

**Additional file 1.** Questionnaire.

## Data Availability

The data used to support the findings of this study are available from corresponding author upon request.

## Abbreviations

BMI: Body mass index
CI: Confidence interval
CR: Creatinine
DBP: Diastolic blood pressure
DKD: Diabetic kidney disease
eGFR: Estimated glomerular filtration rate
FBG: Fasting blood glucose
FINS: Fasting insulin
FT3: Free triiodothyronine
FT4: Free thyroxine
HbA1c: Glycated hemoglobin
HDL-C: High-density lipoprotein cholesterol
HOMA-IR: Homeostasis model assessment of insulin resistance
LDL-C: Low-density lipoprotein cholesterol
MDRD: Modification of diet in renal disease
OR: Odds ratio
SBP: Systolic blood pressure
SE: Standard error
SUA: Serum uric acid
TC: Total cholesterol
TG: Triglycerides
TSH: Tthyroid stimulating hormone
T2DM: Type 2 diabetes mellitus
UA: Uric acid
UACR: Urinary albumin-to-creatinine ratio
WHO: World Health Organization

## References

[1] Cirillo P, Sato W, Reungjui S, Heinig M, Gersch M, Sautin Y (2006). Uric acid, the metabolic syndrome, and renal disease. J Am Soc Nephrol.

[2] Kawamoto R, Katoh T, Ninomiya D, Kumagi T, Abe M, Kohara K (2016). Synergistic association of changes in serum uric acid and triglycerides with changes in insulin resistance after walking exercise in community-dwelling older women. Endocr Res.

[3] Ciarla S, Struglia M, Giorgini P, Striuli R, Necozione S, Properzi G (2014). Serum uric acid levels and metabolic syndrome. Arch Physiol Biochem.

[4] Kodama S, Saito K, Yachi Y, Asumi M, Sugawara A, Totsuka K (2009). Association between serum uric acid and development of type 2 diabetes. Diabetes Care.

[5] Zharikov S, Krotova K, Hu H, Baylis C, Johnson RJ, Block ER (2008). Uric acid decreases NO production and increases arginase activity in cultured pulmonary artery endothelial cells. Am J Phys Cell Phys.

[6] Mazzali M, Kanellis J, Han L, Feng L, Xia YY, Chen Q (2002). Hyperuricemia induces a primary renal arteriolopathy in rats by a blood pressure-independent mechanism. Am J Physiol Ren Physiol.

[7] Talaat KM (2007). el-sheikh AR. The effect of mild hyperuricemia on urinary transforming growth factor beta and the progression of chronic kidney disease. Am J Nephrol.

[8] De Cosmo S, Viazzi F, Pacilli A, Giorda C, Ceriello A, Gentile S (2015). Serum uric acid and risk of CKD in type 2 diabetes. Clin J Am Soc Nephrol.

[9] Jalal DI, Maahs DM, Hovind P, Nakagawa T (2011). Uric acid as a mediator of diabetic nephropathy. Semin Nephrol.

[10] Ruszała A, Wójcik M, Starzyk JB (2019). The impact of thyroid function on the occurrence of metabolic syndrome in obese children and adolescents. Pediatr Endocrinol Diabetes Metab.

[11] Kalra S, Aggarwal S, Khandelwal D (2019). Thyroid dysfunction and type 2 diabetes mellitus: screening strategies and implications for management. Diabetes Ther.

[12] Giordano N, Santacroce C, Mattii G, Geraci S, Amendola A, Gennari C (2001). Hyperuricemia and gout in thyroid endocrine disorders. Clin Exp Rheumatol.

[13] Ashizawa K, Imaizumi M, Usa T, Tominaga T, Sera N, Hida A (2010). Metabolic cardiovascular disease risk factors and their clustering in subclinical hypothyroidism. Clin Endocrinol.

[14] Wang XJ, Qian XW, Zhang X, Han L, Zheng YQ, Wu T (2020). Association of serum uric acid with thyroid function in health check-up participants. Chin Med J.

[15] Ye Y, Gai X, Xie H, Jiao L, Zhang S (2015). Association between serum free thyroxine (FT4) and uric acid levels in populations without overt thyroid dysfunction. Ann Clin Lab Sci.

[16] Perez-Ruiz F, Chinchilla SP, Atxotegi J, Urionagüena I, Herrero-Beites AM, Aniel-Quiroga MA (2015). Increase in thyroid stimulating hormone levels in patients with gout treated with inhibitors of xanthine oxidoreductase. Rheumatol Int.

[17] Expert task force on microvascular complications of diabetes mellitus, Chinese Medical Association (2014). Consensus on prevention and treatment of diabetic kidney disease. Chin J Diabetes Mellitus.

[18] Multi-disciplinary expert task force on hyperuricemia and its related diseases (2017). Chinese multi-disciplinary consensus on the diagnosis and treatment of hyperuricemia and its related diseases. Chin J Intern Med.

[19] McCracken E, Monaghan M, Sreenivasan S (2018). Pathophysiology of the metabolic syndrome. Clin Dermatol.

[20] Yan D, Tu Y, Jiang F, Wang J, Zhang R, Sun X (2015). Uric acid is independently associated with diabetic kidney disease: a cross-sectional study in a Chinese population. PLoS One.

[21] Mauer M, Doria A (2018). Uric acid and diabetic nephropathy risk. Contrib Nephrol.

[22] KDOQI (2007). KDOQI clinical practice guidelines and clinical practice recommendations for diabetes and chronic kidney disease. Am J Kidney Dis.

[23] Raber W, Vukovich T, Vierhapper H (1999). Serum uric acid concentration and thyroid-stimulating-hormone (TSH): results of screening for hyperuricaemia in 2359 consecutive patients with various degrees of thyroid dysfunction. Wien Klin Wochenschr.

[24] Desideri G, Bocale R, D'Amore AM, Carnassale G, Necozione S, Barini A (2020). Thyroid hormones modulate uric acid metabolism in patients with recent onset subclinical hypothyroidism by improving insulin sensitivity. Intern Emerg Med.

[25] Gierach M, Gierach J, Junik R (2020). Insulin resistance and thyroid disorders. Intern Emerg Med.

