# Supplementary material for: Association between decreased thyroid stimulating hormone and hyperuricemia in type 2 diabetic patients with early-stage diabetic kidney disease
Source: BMC Endocr Disord. 2021 Jan 6;21:1. doi: 10.1186/s12902-020-00672-8 (PMC7788793; doi:10.1186/s12902-020-00672-8)
Supplement: Supplementary file 1 — Additional file 1. Questionnaire. [file 12902_2020_672_MOESM1_ESM.docx]

Questionnaire

Case no:

Name:

Sex:

Age:

Height:

Weight:

Blood pressure:

Diabetes: YES NO N/A

Type of diabetes:

Onset time:

Treatment:

Hypertension: YES NO N/A

Onset time:

Treatment:

Primary glomerulonephritis: YES NO N/A

Onset time:

Treatment:

Secondary kidney diseases: YES NO N/A

Primary disease:

Onset time:

Treatment:

Gout: YES NO N/A

Onset time:

Treatment:

[Thyroid diseases](https://fanyi.baidu.com/#en/zh/Thyroid%20Diseases): YES NO N/A

Disease:

Onset time:

Treatment:

Malignancies: YES NO N/A

Disease:

Onset time:

Treatment:

Autoimmune diseases: YES NO N/A

Disease:

Onset time:

Treatment:

Cardiovascular disease: YES NO N/A

Onset time:

Treatment:

Other diseases or drugs:

Onset time:

Treatment:

Alcohol: YES NO

The last time:

Alcohol consumption:
